# Ultrasound-based radiomics analysis for differentiating benign and malignant breast lesions: From static images to CEUS video analysis

**DOI:** 10.3389/fonc.2022.951973

**Published:** 2022-09-16

**Authors:** Jun-Yan Zhu, Han-Lu He, Zi-Mei Lin, Jian-Qiang Zhao, Xiao-Chun Jiang, Zhe-Hao Liang, Xiao-Ping Huang, Hai-Wei Bao, Pin-Tong Huang, Fen Chen

**Affiliations:** ^1^ Department of Ultrasound, The First Affiliated Hospital of Zhejiang Chinese Medical University, Hangzhou, China; ^2^ Ultrasound in Medicine, The Second Affiliated Hospital, Zhejiang University School of Medicine, Hangzhou, China; ^3^ Technology Department, XENIRO, Shanghai, China

**Keywords:** breast cancer, contrast enhanced ultrasonography (CEUS), machine learning, convolutional neural network (CNN), radiomics

## Abstract

**Background:**

Continuous contrast-enhanced ultrasound (CEUS) video is a challenging direction for radiomics research. We aimed to evaluate machine learning (ML) approaches with radiomics combined with the XGBoost model and a convolutional neural network (CNN) for discriminating between benign and malignant lesions in CEUS videos with a duration of more than 1 min.

**Methods:**

We gathered breast CEUS videos of 109 benign and 81 malignant tumors from two centers. Radiomics combined with the XGBoost model and a CNN was used to classify the breast lesions on the CEUS videos. The lesions were manually segmented by one radiologist. Radiomics combined with the XGBoost model was conducted with a variety of data sampling methods. The CNN used pretrained 3D residual network (ResNet) models with 18, 34, 50, and 101 layers. The machine interpretations were compared with prospective interpretations by two radiologists. Breast biopsies or pathological examinations were used as the reference standard. Areas under the receiver operating curves (AUCs) were used to compare the diagnostic performance of the models.

**Results:**

The CNN model achieved the best AUC of 0.84 on the test cohort with the 3D-ResNet-50 model. The radiomics model obtained AUCs between 0.65 and 0.75. Radiologists 1 and 2 had AUCs of 0.75 and 0.70, respectively.

**Conclusions:**

The 3D-ResNet-50 model was superior to the radiomics combined with the XGBoost model in classifying enhanced lesions as benign or malignant on CEUS videos. The CNN model was superior to the radiologists, and the radiomics model performance was close to the performance of the radiologists.

## Introduction

Contrast-enhanced ultrasound (CEUS) is the latest and most important technology in the field of ultrasound imaging (1). Using microbubbles, it obtains detailed information about the tumor blood supply and provides dynamic perfusion information in real time with few application limitations (2, 3). Different from the relatively mature static image radiomics studies, there are technical difficulties in applying static image radiomics analysis methods to video analysis with a length of more than 1 min (4). Given that the practice of medicine is constantly evolving in response to new technology, there is interest in using the latest imaging methods to obtain data for radiomics learning (5, 6).

CEUS is one of the most advanced techniques in clinical tumor treatments, ranging from early screening and differential diagnosis to treatment response evaluation (7). It is particularly useful for the detection and characterization of lesions and has been used in breast cancer diagnosis as a feasible alternative screening modality (1, 8). Breast cancer is the most common malignant cancer in women, and it has a high mortality rate; there were over 1.6 million cases in 2010, and 2.1 million cases are projected by 2030 (9–12). Therefore, early detection and treatment play important roles in reducing mortality rates.

Several studies have reported training a radiomics or convolutional neural network (CNN) architecture to automatically extract features from CEUS cine images. Among them, the application of deep neural networks that can extract continuous spatiotemporal information is quite rare. In this context, we extracted the spatiotemporal information of dynamic CEUS by using 3D-CNN models. We aimed to compare the diagnostic performance of radiomics combined with the ML model, the 3D-CNN model, and human-read interpretations based on CEUS video for the differentiation of benign and malignant breast lesions.

## Materials and methods

### Study design and patients

The study was approved by the ethical committee of the local institutional board and complied with the Declaration of Helsinki. Informed consent was waived for this retrospective research. Written informed consent was obtained from each participant. From April 2021 to November 2021, 123 patients with breast tumors who underwent CEUS examination were enrolled from The First Affiliated Hospital of Zhejiang Chinese Medical University in Hangzhou, China. From August 2018 to August 2021, 92 patients with breast tumors who underwent CEUS examination were enrolled from The Second Affiliated Hospital, Zhejiang University School of Medicine in Hangzhou, China.

The two centers used the same patient inclusion and exclusion criteria. The inclusion criteria were as follows: (a) each patient underwent pathological examination; (b) CEUS examinations were performed before surgery and prior to any treatment, including biopsy or neoadjuvant therapies; and (c) each patient had complete demographic information and clinical data. The exclusion criteria were as follows: (a) poor-quality CEUS cine (e.g., the entire tumor and surrounding breast parenchyma were not clearly displayed on the ultrasound image at the same time) and (b) extreme motion existed during CEUS examination. Finally, the CEUS cines of 190 patients acquired before treatment were analyzed.

**Figure 1:**
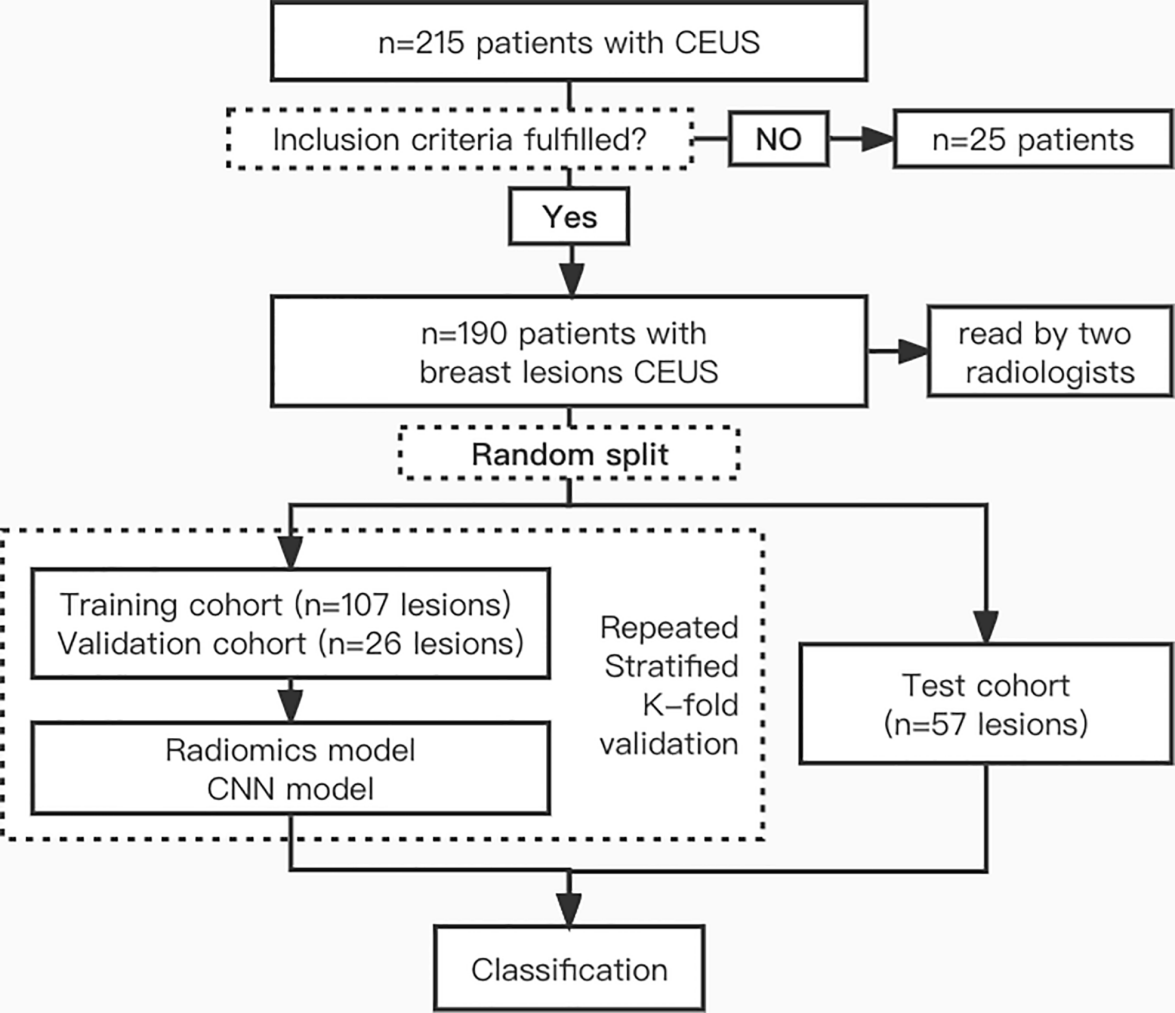
Flowchart of patient enrollment.

There were 190 patient CEUS videos in our dataset, the tumors were classified as benign or malignant, and the dataset included 109 benign and 81 malignant lesions. A detailed flowchart of patient selection for the study is shown in **Figure 1**.

### CEUS data acquisition

CEUS was performed by two experienced radiologists (X-CJ and Z-ML). There were two different ultrasound instruments used in this study (Esaote MyLabTM Twice, Mindray Resona R9).

During the examination, breast tumors were imaged in the transverse plane of the largest tumor dimension. A single focus was always placed at the bottom of the image, and the selected plane remained unchanged. The probe was stabilized manually to ensure that no pressure was exerted to avoid weakening the contrast-enhanced signals. Patient movement was also avoided.

One-minute minimum continuous cine images were acquired after injecting 4.8 ml of the second-generation contrast agent (SonoVue, Bracco Imaging, Milan, Italy) *via* the elbow, followed by a 5-ml saline flush. Time was activated promptly from the beginning of the SonoVue injection.

The selected imaging plane remained unchanged during the examination. The whole CEUS cine process was recorded in an ultrasound workstation by using digital imaging and communication in medicine (DICOM) format. The recorded CEUS videos were used for further analysis.

### Tumor segmentation and preprocessing

Tumors were manually segmented by a single radiologist (FC, with more than 20 years of experience in breast CEUS interpretation). For segmentation, the radiologist first reviewed the complete video to identify tumor boundaries. Then, we extracted a rectangular image with a fixed length–width ratio enclosing the nodule and its surrounding tissues, saved it in JSON format, kept it unchanged in the sequence, adjusted the image size to 160 × 160 pixels, and preprocessed it.

FFmpeg was used to cut the first 10 s and the last 5 s from the dynamic 1.0- to 2.0-min video data. The frame rate of the original data was between 15 and 30 frames/s. We tried to unify it into (15, 18, 21, 24, and 25) different frame rates. After selecting the 18 frames/s video, which had better results, we uniformly converted it into frame pictures.

We used a total of 107 CEUS for the training cohort and 26 CEUS for the validation cohort. A total of 57 CEUS videos were used for the test cohort. We split the dataset using sklearn (version 1.0.2) (13). The train_test_split function with parameter stratifies so that the benign/malignant proportion of training, validation, and test cohorts remains the same as the overall data. The data used in the test cohort were independent and were not used in the training and validation cohorts.

### Radiomics feature extraction and model building

Features were extracted from the ROI using PyRadiomics (version 3.0.1) (14–16). Extracted texture features were calculated on the first-order statistics (19 features), gray-level cooccurrence matrix (24 features), gray-level run-length matrix (16 features), gray-level size-zone matrix (16 features), neighboring gray tone difference matrix (5 features), and gray-level dependence matrix (14 features). A detailed definition of all image features can be found online (http://pyradiomics.readthedocs.io/en/latest/features.html).

The minimum analysis time of a CEUS video is 1 min; a rate of 18 frames/s was applied for a total of 1,080 images. The difference between each picture is small and changes over time. According to expert experience, the degree of change over time should be more valuable for diagnosis. We have tried a variety of data sampling methods: all 60 s of data enter the follow-up; the mean value is taken every 10 s; the mean value is taken every 20 s; one frame is taken every 10 s; one frame is taken every 20 s; the differences between the above values and the 30th second frame are taken; and the differences between the above values and the 60th second frame are taken.

The XGBoost algorithm implements decision trees with a boosted gradient, enhanced performance, and a faster speed. It was applied to the super parameter setting process of gridsearchcv.

### CNN model building

In the training cohort, data augmentation was performed by using random cropping, random rotations, flipping vertically or horizontally, and color jitter (17, 18).

To efficiently utilize the dynamic characteristics of the CEUS modality, multichannel convolution models that can learn the spatiotemporal characteristics of different enhancement models were considered. 3D-ResNet is a subgroup of CNN methods and is widely used in video analysis because it has good performance in dealing with both spatial and temporal features at the same time (19).

Pretrained 3D-ResNet on Kinetics-400 (https://arxiv.org/abs/1705.06950) is used for classification *via* transfer learning (20). To find the most suitable model for benign and malignant discrimination, 18-layer, 34-layer, 50-layer, and 101-layer ResNet models were fine-tuned. Clinical features and lesion size were not included as radiomics and CNN model parameters. The ResNet models were trained by performing Repeat-Stratified K-Fold validation (n_**repeats** = **10**; K = 5) on the dataset to obtain more reliable generalization errors. The dataset was shuffled and equally divided into three cohorts. One was used as the test cohort to evaluate the trained model. Another cohort was further divided into a training set and a validation set according to 80%:20%.

**Figure 2:**
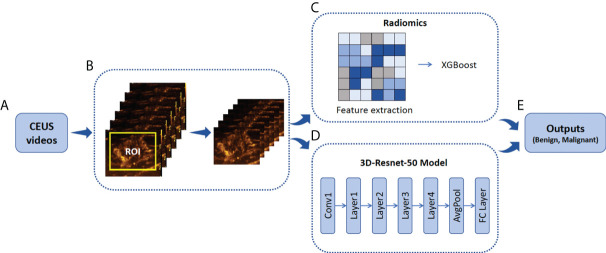
Illustration of ROI annotation in CEUS videos and the design of Radiomics and 3D-Resnet-50 models. **(A)** CEUS examinations were performed for each breast tumor. **(B)** An example of the yellow bounding box ROI drawn in one CEUS frame. **(C)** Schematic of radiomics combined with the XGBoost model. **(D)** A three-dimensional ROI (2D in space and 1D in time) of CEUS videos was fed into the 3D-Resnet-50 model to obtain the discriminative features by automatic feature learning. **(E)** The features obtained from the radiomics and 3D-Resnet-50 models are used to calculate the prediction probability. ROI, region of interest; CEUS, contrast-enhanced ultrasound; ResNet, residual network.

The details of ROI segmentation and the flowchart of radiomics and CNN are shown in **Figure 2**. The following link can be accessed for additional code details: https://github.com/kenshohara/3D-ResNets-PyTorch.

### Reader study

All the digital cine clips of the study population were retrospectively reviewed by two different readers (X-PH and Z-HL, with 8 and 15 years of clinical experience, respectively). The readers were blinded to each other’s interpretations, to the original radiologist’s interpretations, and to the model’s assessment. None of the readers were involved in the CEUS examinations, and both were blinded to the clinical and other imaging information of the patients.

The two readers assessed the possibility of malignancy in CEUS cines based on the Breast Imaging Reporting and Data System (BI-RADS) and the reported BI-RADS categories per patient, which were 3, 4a, 4b, 4c, and 5. Then, the readers combined CEUS, grayscale US, and color Doppler flow imaging (CDFI) for classification in the same way.

### Statistical analysis

All statistical analyses were performed using SPSS (Version 26.0, IBM Corporation, Armonk, USA) and R software (Version 3.4.1, R Foundation for Statistical Computing, Vienna, Austria). Student’s *t*-tests or the Mann–Whitney test, as appropriate, was used to compare continuous variables. The chi-square test was used to compare categorical variables. A *p*-value less than 0.05 was considered statistically significant.

To evaluate the predictions of the three different methods (the radiomics, CNN, and radiologist methods), receiver operating characteristic (ROC) curves were constructed. The areas under the ROC curve (AUCs) with 95% confidence intervals (CIs), sensitivity, specificity, accuracy, and F1 score were investigated.

## Results

### Clinical characteristics

A total of 190 female patients with breast tumors were enrolled for analysis, namely, 109 with benign breast tumors and 81 with malignant tumors, and were divided into two groups. The baseline characteristics of all patients and breast tumors (including age, pathological findings, and US BI-RADS category) are presented in **Table 1**.

**Table 1:** Characteristics of patients and images.

Characteristics	Benign	Malignant	*p*
Patient number (*n*)	109 (57.4%)	81 (42.6%)	
Age (years)	24–78	35–82	<0.05
Range/mean ± SD	45.1 ± 11.2	56.0 ± 10.1	
Size of lesions (cm)	0.32–4.47	0.34–4.54	<0.05
Range/mean ± SD	1.28 ± 0.78	1.97 ± 0.88	
BI-RADS (*n*)			<0.05
3	41 (37.5%)	0 (0.0%)	
4a	49 (45.0%)	13 (16.0%)	
4b	10 (9.2%)	21 (26.0%)	
4c	5 (4.5%)	18 (22.2%)	
5	4 (3.7%)	29 (35.8%)	

SD, standard deviation; BI-RADS, Breast Imaging Reporting and Data System.

The final diagnosis included 109 (57.4%) benign and 81 (42.6%) malignant breast lesions (**Table 2**). Malignant lesions were larger than benign lesions on US, and patients with malignant lesions were significantly older than those with benign lesions. The results showed that the differences between all clinical factors (including age, size, and BI-RADS category) of the patients with benign and malignant tumors were statistically significant (*p* < 0.05).

**Table 2:** Histopathology of breast lesions.

Lesion type	No. of lesions
Benign lesions	109 (57.4%)
Adenosis	32 (29.4%)
Fibroadenoma	28 (25.7%)
Papilloma	15 (13.8%)
Inflammatory process	13 (11.9%)
Other*	21 (19.2%)
Malignant lesions	81 (42.6%)
Invasive	67 (82.7%)
Ductal carcinoma in situ	14 (17.3%)

There were a total of 190 lesions. Unless otherwise indicated, data in parentheses are percentage.

*The “other” category included enhancement around fat necrosis, fresh scar tissue, pseudo angiomatous stromal hyperplasia, and other benign-appearing enhancement because of focal or regional background enhancement.

### Radiologist diagnosis results

**Figure 3:**
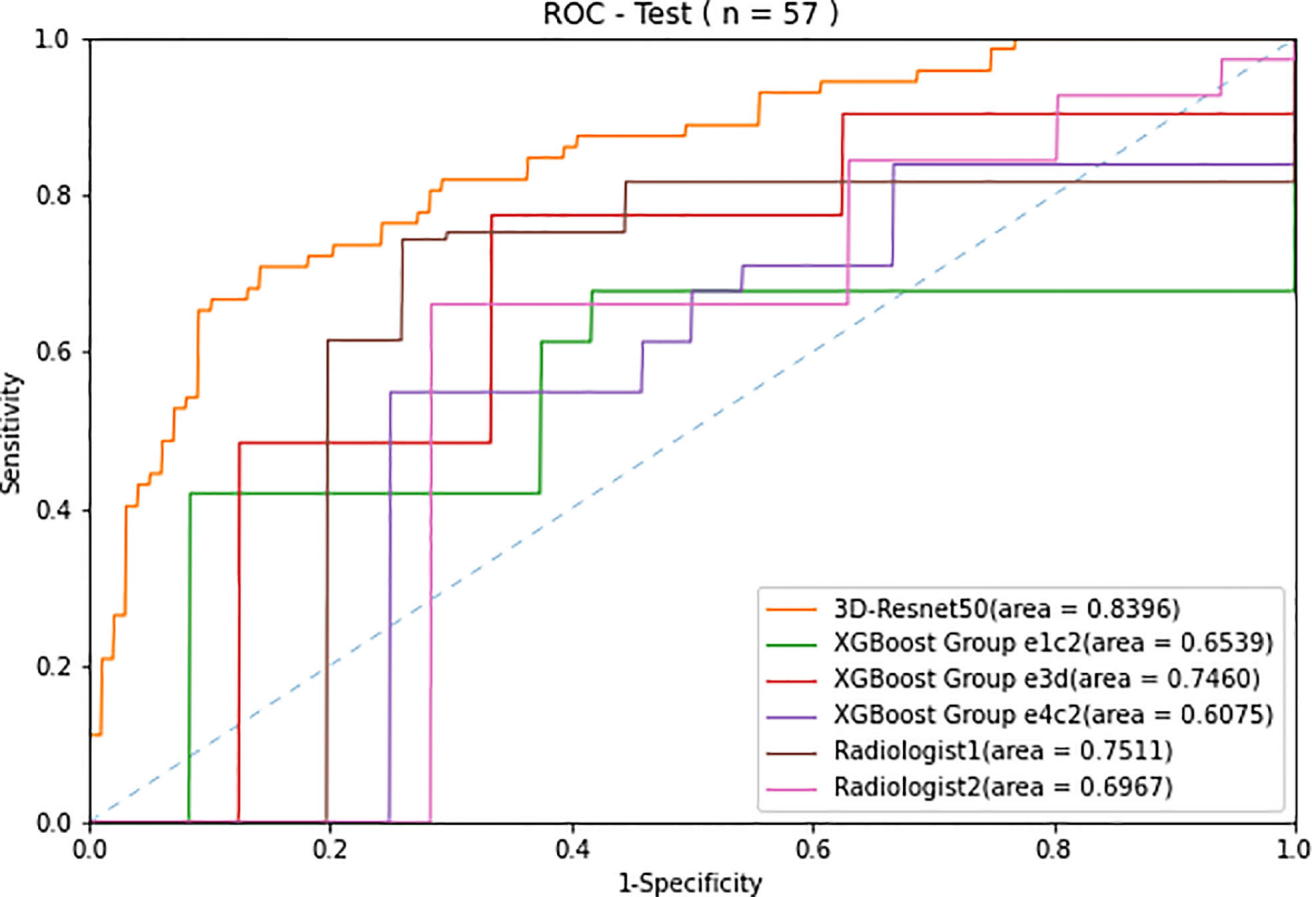
Receiver operating characteristic (ROC) curves of the two radiologists compared with the 3D-Resnet-50 model and radiomics combined with the XGBoost model with three different data sampling methods in the test cohort.

The ROC curves (**Figure 3**) showed that the prediction performance of the senior radiologist (radiologist 1) was better than that of the junior radiologist (radiologist 2), and the AUC values were 0.75 and 0.70, respectively (**Table 3**). Comparing radiologist diagnoses before and after combining CEUS with grayscale US and CDFI, *p*-values for AUC were not statistically significant (*p* > 0.05).

**Table 3:** Comparison of the predictive performance in 3D-Resnet 50, radiomics, and radiologists in the training, validation and test cohort.

Models	Datasets	Sensitivity (%)	Specificity (%)	Accuracy (%)	F1 score	AUC
3D-Resnet 50	Training	83.4	75.7	76.6	0.75	0.84
	Validation	83.4	75.7	75.5	0.74	0.82
	Test	70.8	85.9	76.0	0.72	0.84
XGBoost Group e1c2	Training	72.8	77.6	92.2	0.85	0.98
	Validation	61.8	67.8	69.3	0.92	0.75
	Test	67.7	58.3	54.6	0.55	0.65
XGBoost Group e3d	Training	84.2	79.8	96.7	0.92	0.99
	Validation	65.7	69.5	67.0	0.61	0.74
	Test	77.4	66.7	65.5	0.68	0.75
XGBoost Group e4c2	Training	83.5	84.8	98.7	0.98	1.00
	Validation	64.0	70.9	67.7	0.68	0.74
	Test	54.8	75.0	60.0	0.66	0.61
Radiologist 1	All data	74.3	74.1	74.2	0.77	0.75
Radiologist 2	All data	66.0	71.6	68.4	0.71	0.70

Resnet, residual network; AUC, area under the receiver operating characteristic curve.

### Performance of the radiomics models

The best XGBoost algorithm had the following parameters: learning_rate = 0.02, subsample = 1, min_child_weight = 1, n_estimators = 148, gamma = 0.1, max_depth = 3, and colsample_bytree = 1.

**Figure 4:**
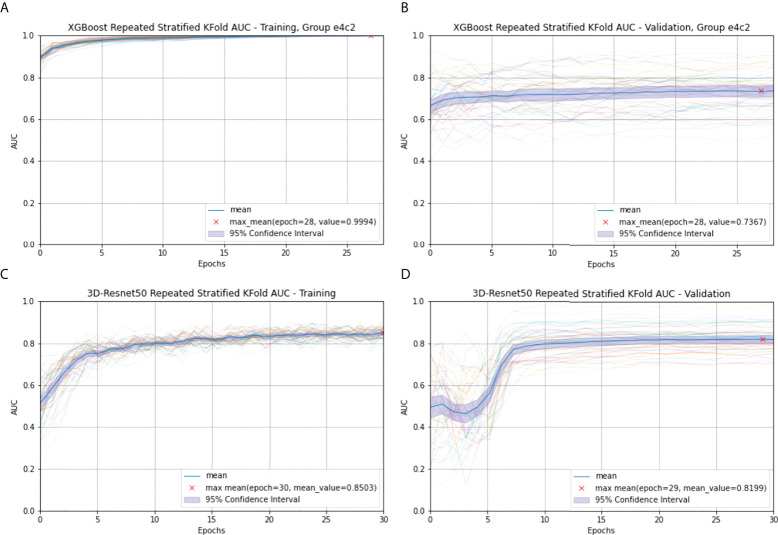
Radiomics **(A)** and 3D-Resnet-50 **(C)** Repeated Stratified 5-Fold AUC in the training cohort; Radiomics **(B)** and 3D-Resnet-50 **(D)** Repeated Stratified 5-Fold AUC in the validation cohort. The epochs are depicted on the *x*-axis, each representing the process of training all training samples once. The thick blue curve represents the mean AUC value. The blue area represents the 95% confidence interval (CI). The red fork represents the maximum mean of the AUC value after repeating multiple epochs.

In the radiomics model of XGBoost Group e4c2, the difference between the frames every 20 s and the frame at the 60th second is taken by using RepeatS Stratified 5-Fold validation in the training and validation cohorts. In the training cohort, after 28 epochs, the max mean AUC was 1.00 (**Figure 4A**). In the validation cohort, after 28 epochs, the max mean AUC was 0.74 (**Figure 4B**).

Among various data packets, the best result is obtained when the differences between the frames at every 10 s and the frame at the 30th second are taken (group e3d, test cohort sensitivity: 77.4%, specificity: 66.7%, accuracy: 65.5%, F1 score: 0.68, AUC: 0.75). The worst result is obtained when the difference between the frames every 20 s and the frame at the 60th second is taken (group e4c2, test cohort sensitivity: 54.8%, specificity: 75.0%, accuracy: 60.0%, F1 score: 0.66, AUC 0.61) (**Table 2**).

### Performance of the 3D-ResNet models

The 3D-ResNet-50 with the best classification effect is selected. The hyperparameters of 3D-ResNet-50 were learning_rate = 1e-4, weight_decay = 1e-5, momentum = 0.9, ft_begin_module = layer1, sample_size = 136, sample_duration = 108, n_epochs = 30, and batch_size = 8. Details of the network architecture are provided in https://github.com/kenshohara/3D-ResNets-PyTorch.

In the 3D-ResNet-50 model, repeat-stratified fivefold validation was used in the training and validation cohorts. In the training cohort, after 30 epochs, the max mean AUC was 0.85 (**Figure 4C**). In the validation cohort, after 29 epochs, the max mean AUC was 0.82 (**Figure 4D**).

In the test cohort, the 3D-ResNet-50 algorithm achieved sensitivity, specificity, accuracy, F1 score, and AUC values of 70.8%, 85.9%, 76.0%, 0.72, and 0.84, respectively. The AUC of the test cohort was the same as that of the training cohort.

### AI system performance compared with the performance of the radiologists

The ROC curves of radiomics, 3D-ResNet-50, and radiologists in the test cohort are shown in **Figure 3**. The AUC of the 3D-ResNet-50 model was 0.84, which was significantly higher than that of both the radiomics and radiologist approaches. The NRI (Net Reclassification Index) of the 3D-ResNet-50 model compared with the radiomics models and radiologists was >0.

The best sensitivity and specificity results for the radiomics model were 77.4% and 66.7%, respectively. The sensitivity and specificity for senior radiologists were 74.3% and 74.1%, respectively. The sensitivity and specificity results for the junior radiologist were 66.0% and 71.6%, respectively (**Table 2**). The NRIs of the best radiomics model compared with the senior radiologist and junior radiologist were <0 and >0, respectively.

**Figure 5:**
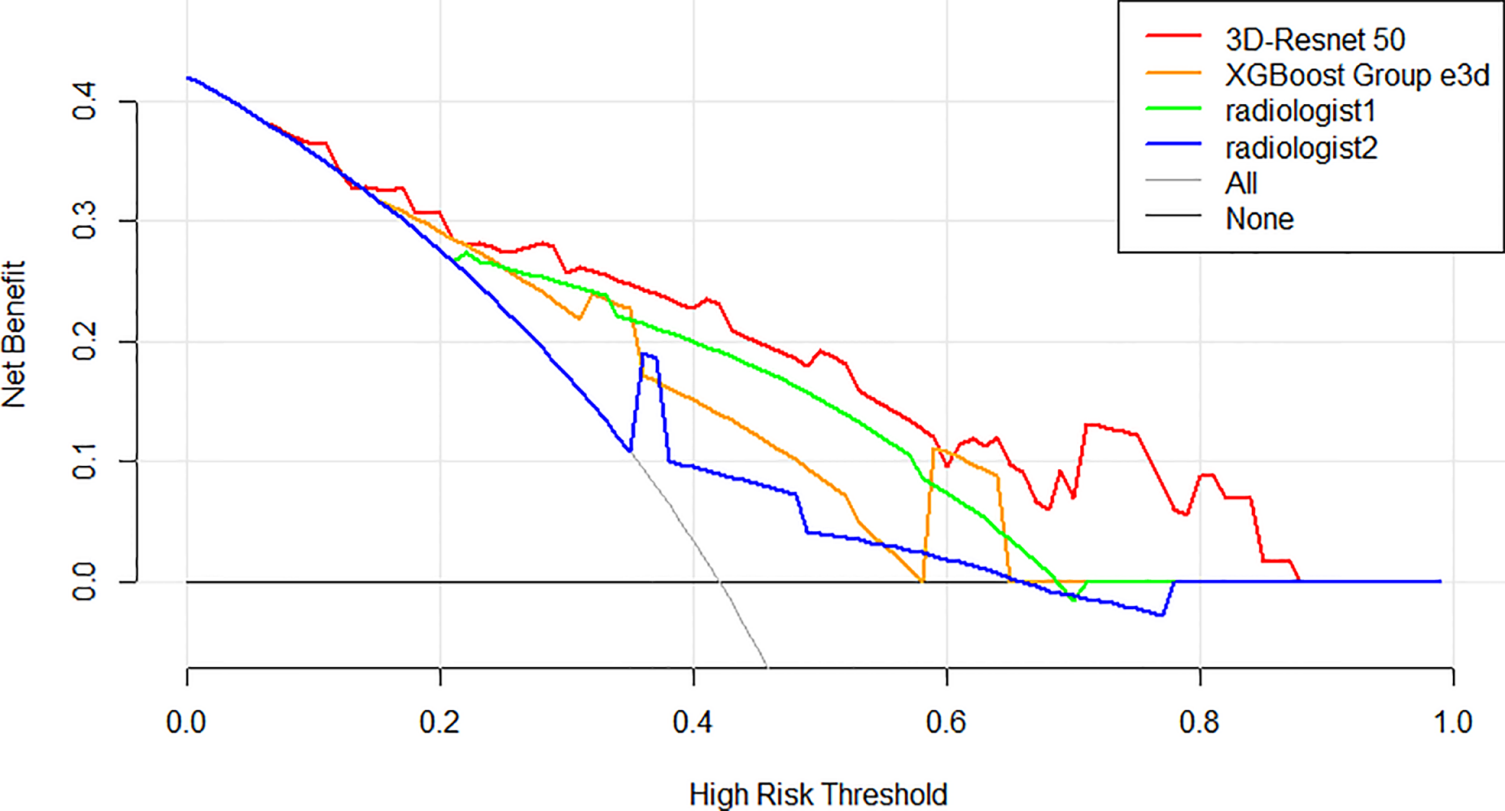
Decision curve analysis (DCA) of the models and radiologists from the test cohort. The net benefit measured on the *y*-axis is determined by calculating the difference between the expected benefit and the expected harm associated with each proposed model. The red curve, green curve, orange curve, and blue curve represent the performance of the 3D-Resnet-50 model, the best XGBoost model, radiologist 1, and radiologist 2, respectively. The gray line represents the assumption that all lesions were malignant (the treat-all scheme). The black line represents the assumption that all lesions were benign (the treat-none scheme). If the threshold probability was more than 7%, using the 3D-Resnet-50 model to predict malignancy added more benefit than either the treat-all scheme or the treat-none scheme (dark black line).

Decision curve analysis (DCA) was used to assess the clinical usefulness of the 3D-ResNet-50 model, radiomics model, and radiologists’ diagnosis in the test cohort (**Figure 5**). If the threshold probability was more than 7%, using the 3D-ResNet-50 model to predict malignancy added more benefit than either the treat-all scheme (assuming that all lesions were malignant) or the treat-none scheme (assuming that all lesions were benign). In addition, using the 3D-ResNet-50 model to predict malignancy added more benefit than using either radiomics or radiologists.

## Discussion

The diameters, volumes, shapes, and contrast-enhanced models of lesions usually described by radiologists based on diagnosis are called semantic features. They can be generated by algorithms that capture imaging data patterns, such as first-order, second-order, and high-order statistical determinants, shape-based features, and fractal features, which are called radiomics features. At present, the research hotspot of image analysis uses CNNs to extract the so-called “deep” feature phase in the training process, which is a very powerful nonlinear mapping. To distinguish CNNs from radiomics algorithms, the medical image task that uses CNNs to extract features is called deep learning radiomics (DL-radiomics) by some scholars. Most radiomics studies use less than 10 images per case, and 1-min CEUS videos have three times more data than static ultrasound images. To our knowledge, this is the first study to attempt to use the above three methods to interpret breast CEUS and evaluate whether CEUS can be used to classify benign and malignant breast lesions.

For the three different methods, we chose a relatively mainstream method combined with the analyzed data. Sufficient time was provided for the semantic analysis of the experts in reading the CEUS. The experts were invited to classify benign and malignant lesions and perform BI-RADS scoring. When processing video for more than 1 min, the conventional radiomics information extraction combined with machine learning classification method must contend with extracting effective information from more than 1,000 times the amount of data from static images. To extract effective information from the massive radiomics data in a video, we referred to the clinical significance (biological underpinnings) and attempted to analyze the difference in the radiomics data at different time points. Data combinations were selected from a variety of time differences. XGBoost, a classical and effective machine learning classification method, was selected, as CNNs can handle a large number of images. We transformed 1 min of video (18 frames/s) into 1,080 pictures, all of which were entered into the follow-up analysis. To obtain the spatiotemporal information of dynamic CEUS and the total amount of data used in this study, we chose to use the 3D-ResNet model for transfer learning.

Our department performs a large number of traditional breast ultrasonography examinations; thus, these radiologists have gained rich experience in interpreting breast ultrasonography images. However, the diagnostic accuracy of radiologists in CEUS video diagnosis is relatively low, with poor CEUS diagnosis consistency among different radiologists, indicating that the human eye has low specificity when observing contrast images in breast lesions (21, 22). Making accurate qualitative cancer diagnoses using ultrasound is still a challenge for radiologists.

The same situation appears in radiomics analysis, which shows that careful selection of image types is very important for obtaining meaningful results. The AUC value we obtained was between 0.65 and 0.75, which is similar to that of human experts. The optimal radiomics analysis in our study involved the expert knowledge of radiologists and data scientists.

Our study with the 3D-ResNet-50 model for predicting breast cancer yielded satisfactory performance, obtaining an AUC of 0.84 on the test cohort. The CEUS-based 3D-ResNet-50 model had excellent performance in identifying benign and malignant breast lesions. Consistent with computer vision trends, CNN spatiotemporal features can help better process video data from CEUS.

In this study, we analyzed the performance of the ML-radiomics method on CEUS videos and found that it exceeded the recognition ability of the human-eye approach. The promising results in this study were attributed to the advantages of the standardized CEUS acquisition criteria and the acquisition of samples from a multicenter database, which created good conditions for our follow-up analysis and made our results more authentic and reliable.

Our work has several limitations.

First, comparing radiologist diagnoses before and after combining CEUS with grayscale US and CDFI, the *p*-values for the AUC were not statistically significant. However, given the scientific nature of research, we should repeat this process for the radiomics and CNN models.

Additionally, a larger cohort of subjects should be included to ensure that the varying perfusion patterns of specific breast lesions can be captured, thus further improving the prediction accuracy and reducing the risk of overfitting.

## Conclusion

Our 3D-ResNet-50 model showed excellent diagnostic performance in differentiating between benign and malignant breast lesions compared with the radiomics combined with the XGBoost model and human readers on CEUS. DL-radiomics may have better results than mathematic-radiomics in the analysis of ultrasonic dynamic images.

## Data availability statement

The original contributions presented in the study are included in the article/supplementary material. Further inquiries can be directed to the corresponding authors.

## Ethics statement

The study was approved by the ethical committee of the local institutional board and complied with the Declaration of Helsinki. Informed consent was waived for this retrospective research.

## Author contributions

Conceptualization: FC. Data curation: H-WB. Formal Analysis: J-YZ, FC. Methodology: J-YZ, FC. Project: P-H. Software: J-YZ. Supervision: Z-HL, X-CJ. Validation: Z-HL, X-PH. Writing – original draft: J-YZ, H-LH. Writing – review and editing: FC. All authors contributed to the article and approved the submitted version.

## Acknowledgments

We thank all participants in this study.

## Conflict of interest

Author J-QZ was employed by XENIRO.

The remaining authors declare that the research was conducted in the absence of any commercial or financial relationships that could be construed as a potential conflict of interest.

## Publisher’s note

All claims expressed in this article are solely those of the authors and do not necessarily represent those of their affiliated organizations, or those of the publisher, the editors and the reviewers. Any product that may be evaluated in this article, or claim that may be made by its manufacturer, is not guaranteed or endorsed by the publisher.
