# Corrigendum: Ultrasound-based radiomics analysis for differentiating benign and malignant breast lesions: from static images to CEUS video analysis

**DOI:** 10.3389/fonc.2024.1436117

**Published:** 2024-06-06

**Authors:** Jun-Yan Zhu, Han-Lu He, Zi-Mei Lin, Jian-Qiang Zhao, Xiao-Chun Jiang, Zhe-Hao Liang, Xiao-Ping Huang, Hai-Wei Bao, Pin-Tong Huang, Fen Chen

**Affiliations:** ^1^ Department of Ultrasound, The First Affiliated Hospital of Zhejiang Chinese Medical University, Hangzhou, China; ^2^ Ultrasound in Medicine, The Second Affiliated Hospital, Zhejiang University School of Medicine, Hangzhou, China; ^3^ Technology Department, XENIRO, Shanghai, China

**Keywords:** breast cancer, contrast enhanced ultrasonography (CEUS), machine learning, convolutional neural network (CNN), radiomics

In the published article, there was an error in the author list, and author Han-Lu He was erroneously excluded as first author. Furthermore, authors Jun-Yan Zhu and Han-Lu He were erroneously included as correspondence. The corrected author list appears below.

Jun-Yan Zhu^1†^, Han-Lu He^1†^, Zi-Mei Lin^2^, Jian-Qiang Zhao^3^, Xiao-Chun Jiang^1^, Zhe-Hao Liang^1^, Xiao-Ping Huang^1^, Hai-Wei Bao^1^, Pin-Tong Huang^2^, Fen Chen^1*^

^†^ Co-first author: Jun-Yan Zhu, Han-Lu He

^*^ Correspondence: Fen Chen

The authors apologize for these errors and state that this does not change the scientific conclusions of the article in any way. The original article has been updated.

